# Parental Understanding and Implementation of Early Peanut Introduction

**DOI:** 10.1001/jamanetworkopen.2025.50915

**Published:** 2025-12-18

**Authors:** Waheeda Samady, Hodan Jibrell, Sara Wasim Malik, Linda Jones Herbert, Corwin Rolling, Ruchi Gupta

**Affiliations:** 1 Ann and Robert H. Lurie Children’s Hospital of Chicago, Chicago, Illinois; 2 Feinberg School of Medicine, Northwestern University, Chicago, Illinois; 3 Center for Food Allergy and Asthma Research, Northwestern University, Chicago, Illinois; 4 Divsion of Psychology & Behavioral Health, Children’s National Hospital, Washington, DC; 5 Department of Pediatrics, George Washington University School of Medicine, Washington, DC

## Abstract

**Importance:**

Early peanut introduction (EPI) is encouraged by National Institute of Allergy and Infectious Disease guidelines and consensus opinion among several allergy associations as a public health measure to decrease peanut allergy. Studies evaluating EPI implementation have found variable impact.

**Objective:**

To comprehensively evaluate parental understanding, beliefs, barriers, and overall implementation of EPI.

**Design, Setting, and Participants:**

For this qualitative study, parents were recruited from primary care academic clinics, federally qualified health centers, and private clinics in Chicago, Illinois, with a focus on adequate representation. Utilizing purposive sampling, all English-speaking parents of infants aged 8 to 13 months were invited to participate. Parents of infants with medical conditions that would alter solid food introduction practices or those involved in other food allergy prevention studies were excluded. Semistructured interviews were conducted with parents from September 2023 to December 2024. Interview questions were structured to follow the Theory of Planned Behavior. All interviews underwent thematic analysis with consensus agreement on all elements.

**Exposure:**

Participation in an interview about EPI.

**Main Outcomes and Measures:**

The primary outcome was EPI understanding and implementation utilizing the following Theory of Planned Behavior domains: knowledge, beliefs and opinions, practices, and information sources. A modified grounded theory approach employing the constant comparison method was used to inform thematic analysis and evaluate interviews.

**Results:**

Forty-nine interviews were conducted. Participants were primarily mothers (45 participants [92%]). Most parents reported they were at least partially aware of EPI guidelines. Eight major themes emerged: (1) EPI was to evaluate whether the infant was allergic to peanut, (2) EPI was to prevent peanut allergy, (3) family history of food allergy was the main risk factor, (4) EPI is viewed positively, (5) fear of an allergic reaction served as a barrier to EPI, (6) parents had mixed feelings about EPI’s overall benefit, (7) EPI was understood and practiced with vide variability, and (8) the pediatrician was the primary source of EPI knowledge.

**Conclusions and Relevance:**

This qualitative study found that although parents were aware of EPI, there was confusion around its purpose, implementation, and risk factors. The main barrier for most parents was the risk of an allergic reaction during EPI. Clear and directive guidance from their pediatrician was a facilitator for successful EPI implementation.

## Key Points

**Question:**

Since the publication of guidelines recommending early peanut introduction (EPI), how do parents of infants perceive, understand, and implement them?

**Findings:**

In this qualitative study, thematic analysis of 49 parent interviews found that although parents were not opposed to EPI, many felt its purpose was to test whether their child had a peanut allergy. This understanding led to variability in when and how EPI was practiced; concerns about severe allergic reaction and lack of belief in EPI prevention serve as barriers.

**Meaning:**

These findings suggest that there is confusion around the purpose, timing, frequency and duration, and risk factors of EPI; education needs to address several barriers, including risk of a severe allergic reaction.

## Teaser

This qualitative study evaluates parental understanding, beliefs, barriers, and overall implementation of early peanut introduction.

## Introduction

The increasing global prevalence of food allergies (FAs) has created challenges at both the population and individual levels,^1^ from burdening the public health infrastructure to impacting the quality of life of affected persons.^2,3^ The most common pediatric FA is peanut allergy (PA), affecting 2% of US children or an estimated 2 children in every classroom.^4^ PA is also associated with the most severe allergic reactions and one of the FAs least likely to be outgrown.^5,6,7,8,9^ However, in 2015, the groundbreaking Learning Early About Peanut trial documented an 81% reduction in the development of PA among their high-risk infant cohort that was introduced to peanut-containing foods at or before age 11 months.^10^ Starting in 2017, recommendations from national health organizations, including the National Institute of Allergy and Infectious Disease,^11^ American Academy of Pediatrics, and several Allergy and Immunology associations,^12^ recommended early peanut introduction (EPI): the practice of feeding infants peanut-containing foods during the early phase of solid food introduction (Box).

Box. **Summary of Current Early Peanut Introduction Guidelines**

**Addendum Guidelines on the Prevention of Peanut Allergy^11^**

Infants with severe eczema, egg allergy, or both have introduction of age-appropriate peanut-containing food as early as age 4 to 6 months to reduce the risk of peanut allergy.
  Evaluation with peanut-specific IgE or skin-prick tests should be strongly considered before introduction.
Infants with mild-to-moderate eczema should have introduction of age-appropriate peanut-containing food around age 6 months.
Infants without eczema or any food allergy have age-appropriate peanut-containing foods freely introduced in the diet together with other solid foods and in accordance with family preferences and cultural practices.
Notes:
  Other solid foods should be introduced before peanut-containing foods to show that the infant is developmentally ready.
  The total amount of peanut protein to be regularly consumed per week should be approximately 6 to 7 g over 3 or more feedings.
  No duration for peanut consumption is specifically noted; however, Learning About Peanut Allergy study outcomes were measured at 12 and 30 months.

**Consensus Approach to the Primary Prevention of Food Allergy^12^^a^**

To prevent peanut and/or egg allergy, both peanut and egg should be introduced around 6 months of life, but not before 4 months.
  Screening before introduction is not required but may be preferred by some families.
Other allergens should be introduced around this time (4-6 months) as well.
Upon introducing complementary foods, infants should be fed a diverse diet, because this may help foster prevention of food allergy.
Notes:
  Consider infants with severe eczema at the highest risk of developing food allergy.
  Consider infants with mild-to-moderate eczema, a family history of atopy in either or both parents, or infants with 1 known food allergy potentially at some increased risk of developing food allergy.
  Be aware that food allergy often develops in infants who have no identifiable risk factors.
  No specific amount or dose recommendations, parents should focus on feeding what their child tolerates with some frequency.
  The recommended duration is several years.

^a^
The 2 other recommendations around hydrolyzed formula and maternal diet are not stated here as they do not pertain to early peanut introduction directly.

Although EPI empowers parents to prevent PA, it also requires them to be aware of the recommendation, embrace a new conceptualization of how allergic disease develops, adhere to recommendations within a specific time window, and risk a potential allergic reaction. A 2021 nationally representative survey of US parents of young children found that fewer than 1 in 5 parents introduced peanuts before age 7 months, and fewer than 3 in 5 did so by age 12 months.^13^ An analysis of free-text survey questions in the Enquiring About Tolerance study found that infant’s developmental readiness, the willingness to eat the assigned foods, and fear of reactions were barriers.^14^ It is unclear what barriers exist in the understanding and implementation of EPI in the general population, and whether and how this public health message is being received by parents.

The objective of this study was to evaluate awareness, beliefs, and practices surrounding EPI using a qualitative approach. In addition, this study explores parental perceptions of EPI in the greater context of solid food introduction, family history of FA, and eczema. Ultimately, understanding these factors will contribute to the development of an informed public health message that can better facilitate EPI.

## Methods

We conducted a qualitative participatory research study that utilized virtual semistructured interviews (30-45 minutes) in addition to a short sociodemographic survey. The 20-question survey asked about race and ethnicity, family structure, parental education, eczema, and family history of FA. We adhered to the Standards for Reporting Qualitative Research (SRQR) reporting guidelines throughout the design, analysis, and reporting phases of this study. First, the study was approved by the institutional review board at the children’s hospital and outpatient network where the study took place. Second, the semistructured interview guide was developed utilizing prestudy focus groups with 24 parents of infants. Third, because there is a deficit in theoretically based research involving EPI, the study and the interview guide questions were also adapted to utilize the Theory of Planned Behavior.^15,16,17,18^ The main outcome measure was EPI-related information based on the Theory of Planned Behavior domains of knowledge, beliefs, behavior control and practices, and norms and information sources. Additional information about this process is provided in eAppendix 1 in Supplement 1.

### Setting and Participants

All English-speaking parents of infants aged 8 to 13 months who were patients at several clinics in the Chicago, Illinois, area were invited to participate. The exclusion criteria included (1) parents of children with medical conditions that would alter solid food introduction practices, and (2) parents involved in other FA prevention studies. These criteria were applied using medical record review and/or the study’s 20-question screening survey. To adhere to SRQR guidelines and obtain data from a diverse group of parents, recruitment occurred at 2 academically affiliated pediatric clinics located in 2 different communities, a private practice network, and a large federally qualified health care center. From September 2023 to December 2024, a total of 431 participants were screened and invited via email and postcard via mail; 105 participants (24%) responded to this initial invitation, with 31 ultimately agreeing to participate. We provided participants with an information sheet, and if they agreed to be interviewed, we took that as informed consent. All potential participants who did not respond to the initial email or postcard were then contacted via telephone call and text message; an additional 29 participants agreed to participate after telephone or text message engagement. Of the 60 participants who were scheduled for an interview, 49 (83%) completed the study (eFigure and eAppendix 1 in Supplement 1).

### Interview Procedures

Interviews were conducted via Zoom by an investigator who was trained in qualitative methods (W.S.) and were recorded, transcribed verbatim, and coded independently by 2 investigators (W.S. and H.J.) using MaxQDA version 24.7 (VERBI Software). The initial interview codebook was developed from the interview guide. This codebook was applied to the first 5 interviews; any differences in coding were identified and discussed, and adjustments were made to coding approach. This finalized codebook was then applied to all subsequent interviews, and any differences with codes were discussed and converged until the team reached agreement (consensus agreement).

### Data Analysis

To increase trustworthiness of data analyses per SRQR guidelines, the analysis team included a pediatrician and 2 public health researchers (W.S., H.J., and S.W.M.), who reviewed interviews independently first with documented notes and then the team met to discuss themes. A modified grounded theory approach employing the constant comparison method was used.^19^ Major and minor themes were inductively extracted and established by consensus. After 35 interviews, saturation was reached for several major themes (eTable in Supplement 1). However, single parent households and children with Medicaid were not adequately represented. Thus, a targeted recruitment of parents who met one or both criteria (using medical record review and survey) was conducted to invite more parents from these groups. Recruitment continued until thematic analysis found saturation was reached in all demographic groups (at 49 interviews). A subanalysis evaluating several variables including private or public insurance, parental education, and family structure was conducted. These procedures are further described in eAppendix 1 in Supplement 1.

## Results

Table 1 describes demographic characteristics of our sample of 49 interviews; participants were primarily mothers (45 participants [92%]) with the following racial and ethnic groups represented: 2% American Indian or Alaska Native (1 participant), 8% Asian (4 participants), 29% Black (29 participants), 29% Hispanic or Latino (14 participants), and 43% White (21 participants). Eight major themes and 7 minor themes emerged from the interview data; in line with SRQR guidelines, deidentified quotations for all themes are provided in Table 2 and Table 3.

**Table 1.** Demographics of Parents and Caregivers Participating in the Study

Characteristics	Participants, No. (%) (N = 49)
Relationship to child	
Mother	45 (92)
Father	2 (4)
Couples	2 (4)
Insurance type	
Medicaid	28 (57)
Private	21 (43)
Race	
American Indian or Alaska Native	1 (2)
Asian	4 (8)
Black	14 (29)
White	21 (43)
Multiracial or other	7 (14)
Unknown or not disclosed	2 (4)
Ethnicity	
Hispanic or Latino	14 (29)
Not Hispanic or Latino	34 (69)
Unknown or not disclosed	1 (2)
Education	
Less than a bachelor’s degree	18 (37)
Bachelor’s degree or higher	25 (51)
Graduate degree	6 (12)
Family structure	
2-Parent household	41 (84)
Single-parent household	8 (16)
No. of children	
1	21 (43)
≥2	28 (57)
Family history of food allergy	
Yes	12 (24)
Unsure	1 (<1)
Infant with eczema (parent-report)	
Yes	11 (22)
Unsure	3 (6)

**Table 2.** Major Themes and Example Quotations

Major theme (domain)	Quotation (interview No.)
Major theme No. 1: EPI was to prevent peanut allergy (knowledge)	“Yeah. I really wanted to be mindful of certain allergens. I really didn’t want her to have a peanut allergy, so I wanted to introduce it early on and introduce it often to make sure that she doesn’t become allergic to peanuts.” (Interview 39) “...And so for us, it made a lot of sense that just early exposure, frequent exposure would decrease the risk for food allergies. So that’s why we were very much on board.” (Interview 28) “I mean, that’s probably a way of their body just getting used to it, like those nutrients and stuff, and that’s how they also start growing up and their body starts recognizing stuff and that wanting to attack it if you could put it that way.” (Interview 29) “I see it makes sense because early introduction will lower the risk of getting allergy.” (Interview 45)
Major theme No. 2: EPI evaluates whether the infant is allergic to peanut (knowledge)	“I think it makes sense because, I mean, if they’re gonna be allergic, they’re gonna be allergic. I mean, it’s still like no matter the age. So why wait?” (Interview 21) “I think that ruled out the worry of, will she be allergic, will she not be allergic and then her being exposed to it at a different place where you’re not even aware or with other people. It did give me a little bit of that control of being able to produce it….” (Interview 2) “I believe they were saying that I should introduce peanut butter to him just to know if he has a food allergy to it. So, that’s something that we know how to handle in the future just in case he has an allergic reaction.” (Interview 26) “She [the pediatrician] told me that I could start introducing, just to see what his reaction would be. To start trying to give him peanut butter.” (Interview 9) “I spoke with his pediatrician at about six months. She was saying to see if he’s allergic to peanut butter.” (Interview 4)
Major theme No. 3: family history of food allergy was the main risk factor (knowledge)	“I don’t think I was really concerned only because no one in my family has any allergies. I didn’t have any allergies.” (Interview 2) “No, I wasn’t really scared. I was just kind of like—because nobody in my family or her dad’s family has a peanut allergy….” (Interview 22) “Well, compared to the history of my boyfriend and mine, none of us has been an allergic. Honestly, I have no worries or anything like that.” (Interview 13) “But we don’t have like, a lot of food allergies in our family to begin with. So we weren’t super concerned about it.” (Interview 6)
Major theme No. 4: EPI is viewed positively (opinions and beliefs)	“I thought it was easy. I didn’t feel so scared. I didn’t feel so nervous about that. I don’t want them to have allergies to regular food thing, everyday things. So, I was like, we should start, we should do it.” (Interview 1) “I agree with it. I think they should be incorporated with these foods to get their bodies used to them. Allergies are really interesting.” (Interview 18) “That made sense.... Yeah, because if not, I probably wouldn’t have made it and she wouldn’t have been exposed to it. And it’s just, it’s a lot easier to know now that she’s okay with it than later.” (Interview 22) “So, this is a good news. So if it prevents the allergic, first, we check when we give him. I don’t think he has any allergy. But if peanut butter protects from allergy, we’re happy to hear that.” (Interview 23)
Major theme No. 5: fear of an allergic reaction served as a barrier to EPI (opinions and beliefs)	“I have steered away from peanut butter altogether so far…I do think that I probably will introduce them to peanut butter like once they get past that one-year mark. I’ve just been a little hesitant with that because if there is any peanut allergy, I know the risk of their throat closing and stuff like that because it makes me frantic.” (Interview 19) “So, I was just trying to ease that into it, because I’m a first-time mom and I don’t want to freak out from a reaction or who knows how bad the reaction can be, because it’s not always hives.” (Interview 15) “Yeah, I was really nervous to give her anything, like, any allergens. Because I was like, what if she’s allergic, what do I do? Because I’m really scared...I make sure I have my keys and everything near me just in case I had to rush her to the hospital....” (Interview 2 “I’ve been very hesitant because I know that’s a very common allergy nowadays with all the kids. In my head, I wanted to do it…[but] I wanted to go to the park and be next to the hospital.” (Interview 13) “That’s why when they told me to first introduce it to her, it took me so long. So, when I went back for her six-month checkup, they were like, ‘So, did you introduce peanut butter to her?’ And I’m like, ‘No, actually I didn’t. I’m kind of scared.” (Interview 8)
Major theme No. 6: parents had mixed feelings about EPI’s overall benefit (opinions and beliefs)	“It could have prevented a food allergy. It seems like that’s what research says. I can’t know that either way. And the benefits she ate food, filled her tummy. She seems to like it.” (Interview 42) “Possibly, I don’t know. I can’t say, I think she would for sure have an allergy if she didn’t have it. Like I told you earlier we don’t have that many food allergies in our family.” (Interview 6) “I say yes and no because I feel like...they probably told my mom the same thing and my sister ended up getting allergic reaction when she get older. So, I say like I feel like it’s really not but it’s just like what they think is best.” (Interview 11) “I always feel like everything can change. There is many of my friends that when they eat, they’re like, oh my God, I’m allergic to this. I’m allergic to certain stuff after being pregnant. So, things can change.” (Interview 13) “I think in the grand scheme of things, I’m not sure how much I was able to prevent her being allergic to foods as an adult, but I also knew it wouldn’t hurt her to expose her early.” (Interview 30)
Major theme No. 7: EPI was understood and practiced with vide variability (practices)	“We had a system.... So, we did the yogurt and the yogurt went well, that first week. We did it Monday, Wednesday, Friday. And then, the next week we did it with the peanut butter, Monday, Wednesday, Friday mixed in with the yogurt.” (Interview 10) “So, I did the baby spoons. I did like a half of like the little dip in there and just gave it to them with a spoon. We did that a couple of times, maybe like once a week for like a month or two. And then after that we kind of stopped.” (Interview 30) “I did not keep it up, I just gave it to him. Kinda like one and done and that’s been it.” (Interview 4) “I would say, oh, it just depends. Maybe two to three times a week. It just depends on what he’s having for breakfast, but I do try to include it pretty often just so he is pretty used to it.” (Interview 29) “I think it’s just like, whenever it’s out it’s, but we don’t have strict diets in our house, like, oh, if her brother is eating a peanut butter sandwich and she’s sitting there, we’ll give her a little spoonful of peanut butter to try to eat.” (Interview 6) “So it was two tablespoons of peanut butter mixed with hot water.” (Interview 12)
Major theme No. 8: the pediatrician was the primary source of EPI knowledge (sources of information)	“...And then when I went to the doctor, they said like, ‘Oh, try to introduce him to all of the allergens right away so you can figure that out.’ So, I just slowly added like peanut, egg, fish, seafood. That’s kind of how I did it. Like why I did it the way I did it is just because the doctor said that’s the best way....” (Interview 16) “And I feel like it was the reassurance of a doctor telling me like there’s nothing to be afraid of. She’s young at this point.” (Interview 8) “Yeah, I like that my doctor was like, yeah, you should do it just to make sure, just giving me information and reassuring me that what I’m doing is right and letting me know that it’ll help her in the long run, because that’s why I wanted to give her everything.” (Interview 24) “So I was kind of iffy about introducing those. Also with peanut butter, I kind of delayed it a little. But we have introduced them. I talked to the doctor and she kind of guided me as to how to do it and so far, no allergies.” (Interview 3)

Abbreviation: EPI, early peanut introduction.

**Table 3.** Minor Themes and Example Quotations

Theme	Quotation (interview No.)
Major theme No. 4: parents had a positive perception of EPI (opinions and beliefs)	
Minor theme: EPI was less important to families who disliked peanut products and/or not worried about food allergies	”No, I have not. I, myself do not have any allergy to peanuts. And I even didn’t know that if the peanuts is good for 11-mo-old baby. I thought it’s maybe a little too early for the baby. So, I never thought about peanuts.” (Interview 5) “But I haven’t—I don’t think I’ve given him peanut butter yet. I just didn’t focus on doing that. I guess that would be in the future. My husband hates peanut butter. So, maybe that’s why it’s not in our house.” (Interview 18)
Major theme No. 6: parents were unsure whether they believed EPI was advantageous (opinions and beliefs)	
Minor theme: parents want to know the science	“And so, I’m always looking for evidence-based research and not necessarily, what’s mainstream.” (Interview 10) “So, if somebody told me we’ve like done studies and it is proven or look good that she’ll be less likely to have an allergy if she has these foods before six months, then definitely we would do it.” (Interview 14) “And let’s say, if I am allergic to wheat and if I was exposed to wheat when I was a baby, that alleviate the allergy issue, I don’t know? And I need to know about more scientific background or maybe backup for that.” (Interview 5)
Minor theme: awareness about previous guidelines made parents more skeptical	“I was a little bit shocked, because I actually have a 12 year old. He is 12 years apart with (current child). So back when he was small and he was a baby, I remember that I was told try to avoid it until he’s like bigger, because you don’t know if he’s going to have a reaction, and it was avoided until after he was one is when we introduced him to peanut butter, we introduced him to eggs. I was a little bit shocked to hear that guidelines were changed.” (Interview 17) “I have a 16 and a 13-year-old.... I didn’t give them that stuff until they were a little more active as far as like their motor skills and able to tell me or show me that something is wrong. So that’s just my preference. Things could be different now, I’m just a little hesitant on giving him peanut butter.” (Interview 9)
Major theme No. 7: there was significant variation in the amount, the frequency, and duration in which peanut product was given (practices)	
Minor theme: EPI was easier if peanuts were a family food	“My husband eats a lot of nuts and they put a lot of peanuts in their foods from their culture. I’m like, okay, since you guys eat it and then it is an allergen…I should like give it to her.” (Interview 24) “I want her to enjoy. I love peanut butter, and I want her to eat it with me.” (Interview 27) “I really love peanut butter, and I eat it probably every day. So I had asked my doctor about—I have friends with children with peanut allergies, and she was like, well, one of the best ways they’re finding to help that is to early expose and expose a lot.” (Interview 47)
Minor theme: EPI facilitated by incorporating in infants’ meals	“I buy peanut butter crackers all the time because that’s my other kids snack of choice. They like peanut butter crackers.” (Interview 20) “Yeah, it went really well, because that’s what we eat for breakfast every morning, yogurt and peanut butter with bananas.” (Interview 24) “It became part of her diet. Yeah. So I also think, because like I mentioned, it makes it such a balanced snack to add the peanut butter, and it also just is so easy. It definitely became, like a normal thing. And we also always have almond butter and peanut butter in the house. So, it was easy.” (Interview 40)
Major theme No. 8: the primary source of information around EPI was the pediatrician (sources of information)	
Minor theme: parents trust pediatrician over other sources of information	“And I remember my friend, her baby is three years old and she didn’t give him eggs until he was one. So, I was like, ‘Oh, I have to do it like that too.’ But then the doctor was like, ‘No, the longer you wait, the more allergies they could have.’ And we did it sooner and they didn’t have any allergies, luckily.” (Interview 1) “They have these meal plans and they’re like, introduce this food, then this food, then that food, and they have this whole thing about how they have this research about how you don’t have to wait. But then obviously at the pediatrician, they want us to wait. And so we opted and defaulted to the pediatrician’s advice.” (Interview 28) “It was my main source because I would go online and then I also (specific) book that I referred to a lot. I asked his doctor a lot of questions. It’s kind of 50/50. I would read it and then at his check-up appointments for his next phase, I would say, okay, I want to think about doing this. Can I do this? So I took a lot of their advice to heart and I think the very last resource in desperation was outside information....” (Interview 7)
Minor theme: directive guidance from their pediatrician facilitated correct implementation	“I was in the clinic. I think when she—when it was the six-month appointment, he said she mentioned, ‘Have you introduced peanuts or to the baby?’ And I said, ‘No.’ She said, ‘Would you feel comfortable? This is how I would suggest you introduce it.’ And then after we had that conversation, I went home and tried it out.” (Interview 3) “We got the list of the, like, Big-8 or Big-9 from the pediatrician at our six-month appointment. And it was recommended to introduce these as early as possible, wait 3 or 4 days between allergen introductions, and write down the first and second time, because we were told that the reaction to allergens can actually be more likely at the second exposure.” (Interview 42)

Abbreviation: EPI, early peanut introduction.

### Themes by Domain

#### Awareness and Knowledge

There were variable levels of awareness regarding the EPI guidelines, with the majority of parents being somewhat aware (40 parents [82%]); some explicitly understood they were specific recommendations to reduce PA, whereas others were only generally aware of EPI. Regarding knowledge, some parents thought that EPI’s primary purpose was to prevent PA; others thought it was to evaluate whether their infant would react to peanut in a controlled environment. A few parents expressed belief in both of these purposes. Regarding risks for FA development, most parents thought that family history of FA was the main risk factor for developing PA. Very few understood that having atopic dermatitis (AD) or eczema conferred any risk. Notably, this was true for both those who did and did not have infants with AD.

#### Perceptions, Opinions, and Beliefs

Many parents who were aware of EPI reported a positive perception of the practice. Many felt empowered by the guidelines that helped prevent an allergy and/or control the situation in which their infant is exposed to an allergen. However, there were some parents in the study who did not care for EPI and they were not concerned about PA, either because it was not an issue in their family or because peanut products were disliked by adults in the family. In addition, a few parents who had older children still remembered the recommendations about delaying peanut products and were wary of the new guidelines.

Even among those who had knowledge and a positive perception of the practice, some expressed fear of a severe allergic reaction when considering EPI. The parents who were able to overcome this fear employed a variety of strategies that helped them facilitate EPI, such as assuming their infant would not react because there was a lack of FA in their family or doing their own research on the safety of EPI (cognitive facilitators). Others enlisted support from family and friends who had already practiced it or began EPI when more than 1 caregiver was present (social support facilitators), whereas others ensured they had appropriate medications on hand (epinephrine and antihistamines) or were close to a medical facility when starting EPI (medical facilitators). Some parents were also motivated to overcome their fear of peanut introduction because peanut products were common in their family or cultures, or they thought they were a healthy source of nutrition for their infants (diet facilitators).

In response to whether they believed EPI was beneficial, parent responses were variable. A few parents were very enthusiastic about its advantages, whereas others were dismissive of any benefits. However, the majority responded with mixed feelings about EPI, expressing they did not know whether there was any true benefit but also thought it was unlikely to cause any substantial harm. This lack of confidence in the benefit of EPI existed even among those who had expressed positivity about EPI and followed the guidance. Some did not believe EPI could really work because they knew people who developed adult-onset FA, whereas other parents stated that they would believe in the guidelines more if they knew the science behind it.

#### Behavior Control and EPI Practices

In our sample, most parents had given peanut products to their infant at some point at less than 12 months of age; however, there were some who expressed they were delaying until after age 1 year. In the subset of our participants who gave peanut, some parents participated in EPI at 6 months, coming home from their infant’s 6-month visit and giving peanut products that day. Others gave peanut-containing products at some point in the first few months of taking solid foods, whereas others wanted to start as early as possible (ie, as soon as their infant could tolerate any solid foods, approximately 3-4 months). The timing of EPI and the urgency around it was related to parental understanding its purpose, but also family history of FA, and having a peanut product readily available (eg, if the parent already had a peanut butter brand they approved of or if peanuts were a common family food).

Guidelines around EPI state that after their introduction, peanut products should be given regularly (Box). Our analysis found there was wide variation in the amount of peanut product given, its frequency, and duration. Some parents thought a small amount (ie, “a dip of peanut butter”) was adequate exposure to peanut protein, whereas for others it was a starting place, and the amount of peanut product was subsequently increased on future feedings. Others reported following specific handouts from their pediatrician in which 1 to 2 tablespoons of peanut butter was given.

After this initial peanut feeding, some parents never gave peanut product again (“one and done”), some gave it a few more times, and others continued to incorporate it into the infant’s weekly diet over the course of several months. EPI frequency and duration were connected to many factors, including the infant’s like or dislike of the peanut product, the parent’s understanding of and commitment to EPI, and whether peanut-containing foods were common in the family. For example, parents who believed that early peanut introduction was solely to see whether their infant was allergic may not keep peanut products in the diet unless it was a family food. We found that parents who kept peanut products in their infant’s diet facilitated this practice by intentionally incorporating peanut protein into one of the infant’s meals, such as making it a breakfast food or an after-nap snack, or sharing peanut product with their infant when it was eaten by other members of the family (eg, an older sibling).

#### Sources of Information and Norms

Most parents who endorsed EPI awareness reported that their pediatrician was the main source of EPI information. Other sources of information included social media, podcasts, websites or smartphone applications regarding infant nutrition, or word-of-mouth from family and friends. Even among those parents who learned about EPI outside of their pediatrician’s office, many still sought more information from their pediatrician. Parents also reported trusting their pediatrician’s guidance if it conflicted with online sources and wanted their pediatrician to help them understand what resources were trustworthy. Furthermore, we found that those who reported that they followed the EPI guidelines correctly according to timing, dose, and frequency were those who were given clear and directive guidance from their pediatrician. However, there were still some parents who were unaware of EPI, reporting they had never heard of it from their pediatrician or other sources. Additional results are shown in eAppendix 2 in Supplement 1.

## Discussion

Several studies have evaluated EPI impact; however, to our knowledge this is the first study to use qualitative interviews to evaluate parents’ EPI awareness, knowledge, and perceptions. Previously, a national survey of US parents found 47.7% believed that feeding peanuts early prevented PA, and yet only 17.2% first offered peanut-containing foods before age 7 months.^13^ In Australia, large cross-sectional studies found their EPI guidelines were not associated with a statistically significant decrease in PA prevalence. They also found that EPI varied according to ethnic and racial groups, suggesting that differing introduction patterns and beliefs may have contributed to the findings.^20^ However, another study found when Australian families were given focused education and practical tips on how to incorporate allergenic foods in their infants’ diet at 6 months, peanut and egg were introduced at approximately age 6 months by more than 94% of families, and PA and egg allergy rates decreased significantly (from 5.8% to 1.1% for PA and from 11.7% to 2.8% for egg allergy; *P* < .001).^21^ The Enquiring About Tolerance study also analyzed parental feeding problems in their intervention group, finding infant refusal, reaction fears, and lifestyle limitations were barriers.^14^ These studies provide some perspective around guideline compliance, yet several gaps remain. This analysis offers an in-depth description of factors that affect EPI, how information shapes beliefs, how beliefs affect practices, and how different barriers and facilitators interact to create varying levels of compliance.

The reversal of previous recommendations advising against peanut introduction^22,23,24^ did not present a barrier for most families in our sample, except those with older children. As time progresses, this shift is likely to be less of a barrier. Currently, most parents describe EPI as a positive and empowering practice, and many reporting it “makes sense.” However, these positive beliefs are then countered by challenges such as finding a suitable peanut product and EPI planning. Addressing these issues could involve providing peanut products in clinics or offering lists of appropriate products available in the community.

Similar to other studies,^14,24,25,26^ fear of severe allergic reactions was a common concern for many parents considering EPI. Although some continued with the practice despite these fears, others chose to delay peanut introduction until their child was older. Even among participants who proceeded with EPI, this fear contributed to delays in planning the introduction. To better support these parents, education on the actual incidence of allergic reactions and guidance on how to recognize and respond to them is essential. On the basis of parent interviews, we suggest starting EPI while their child is healthy, and beginning with small amounts, as a way to reduce this anxiety. Offering an initial peanut feeding in a pediatric office or via telehealth could further support high-risk infants and/or parents who want more support.

AD is the most important risk factor for food allergies,^27^ but many parents were unaware of this connection, regardless of their child’s AD status. The National Institute of Allergy and Infectious Disease guidelines recommend earlier peanut introduction for infants with moderate-to-severe AD after testing, and the consensus guidelines emphasize the correlation between AD and FA development (Box).^11,12^ Ideally, AD management, which reduces skin exposure to food proteins, should go hand-in-hand with EPI to reduce FA development.^28,29^ Public health messaging needs to better highlight AD as a risk factor and encourage families of infants with AD to successfully introduce and maintain peanut products in their diet.

Our study also identified other areas for improvement for EPI public health messaging. Misunderstandings existed around the primary purpose of EPI and its implementation (ie, timing, dose, frequency, and duration). Although identifying a PA early can be beneficial, the goal of EPI is to prevent PA, and encouraging earlier introduction (<7 months) would maximize the prevention effect.^29,30^ Notably, the wide variations in how long peanut products remained in the child’s diet may be directly reflective of the guidelines not clearly stating that peanut consumption should be maintained through infancy and into toddlerhood. Other studies have also commented on how substantial recommendation variability can cause confusion for parents.^31^ More education about how to keep peanut products in the diet is needed to make EPI successful and address questions around adult-onset allergy.^32^

Similar to findings from the national parent survey,^13^ we found the role of the pediatrician is central in supporting EPI. Given the time constraints during well-child visits, educating parents about EPI in depth is challenging. In addition, addressing the role of AD as a primary risk factor for FA development and empowering parents with this knowledge—rather than instilling fear—is critical. As such, pediatricians must be supported to provide clear, actionable guidance on when, how, and how often to introduce peanut products. This education should be complemented by parent handouts, videos, and online content.

### Limitations

This study has several limitations. It was conducted in a large, urban center, so the findings may not fully represent families in suburban or rural settings or the broader US. However, many of the findings were consistent with the national parent survey, including differences in guideline awareness and the role of pediatricians. Our sample had a higher proportion of college-educated parents than the general population, but one-third lacked a bachelor’s degree, ensuring a broader representation. In addition, our study was conducted in English with English-speaking participants. Although our sample was racially and ethnically diverse and included participants who spoke other languages, it may not reflect the perspectives of non–English-speaking families.

## Conclusions

In this qualitative study, we found that although many parents were aware of EPI, there is confusion around its purpose, timing, frequency, and dose and the risk factors for PA development. These are important aspects of the public health message that need to be improved. The greatest facilitator for successful early peanut introduction was clear guidance from their pediatrician.

## Notes

Supplement 1. eAppendix 1. Supplemental Methods eFigure. Recruitment Flow Diagram eTable. Quantification of Major Themes eAppendix 2. Supplemental Results eReferences

Supplement 2. Data Sharing Statement
