# Supplementary material for: Parental Understanding and Implementation of Early Peanut Introduction
Source: JAMA Netw Open. 2025 Dec 18;8(12):e2550915. doi: 10.1001/jamanetworkopen.2025.50915 (PMC12715651; doi:10.1001/jamanetworkopen.2025.50915)
Supplement: Supplement 1. — eAppendix 1. Supplemental Methods eFigure. Recruitment Flow Diagram eTable. Quantification of Major Themes eAppendix 2. Supplemental Results eReferences [file jamanetwopen-e2550915-s001.pdf]

Supplemental Online Content

Samady W, Jibrell H, Malik SW, Herbert JW, Rolling C Jr, Gupta R. Parental understanding and implementation of early peanut introduction. *JAMA Netw Open*. 2025;8(12):e2550915. doi:10.1001/jamanetworkopen.2025.50915

eAppendix 1. Supplemental Methods

eFigure. Recruitment Flow Diagram

eTable. Quantification of Major Themes

eAppendix 2. Supplemental Results

eReferences

This supplemental material has been provided by the authors to give readers additional information about their work.

© 2025 Samady W et al. *JAMA Network Open*.

eAppendix 1. Supplemental Methods

Pre-study Focus Groups

Several focus groups with parents of children aged 8-16 months were conducted; several versions of questions and probes were field tested to determine how they were interpreted by parents and what types of information they elicited. It was also determined that age >13 months resulted in challenges with infant feeding recall, which was used to establish the age inclusion criteria for the study.

Theory of Planned Behavior

TPB is a well-validated decision-making model that identifies the key determinants of a behavior (such as EPPI). The TPB explores the psychosocial influences on behavior and provides an appropriate construct for identifying parents' attitudes, normative influences toward, and perceived behavioral control of EPPI. Previous studies based on the TPB have successfully identified modifiable factors in the areas of parenting, child health, and nutrition.[1-4]

Final Interview Guide Questions pertaining to EPPI

Recently there have been recommendations to feed your baby peanut products (things like peanut butter) as a way to prevent peanut allergy; did you hear about these guidelines? [Knowledge]

If participant answers YES:

1. What did you think about them when you first heard of them? [Beliefs/Opinions]
2. Where did you hear about this information? [Sources of Information]
3. What were you told about giving peanut butter? [Sources of Information]
4. What was your understanding of why you should give it? [Knowledge]
5. Did you decide to feed your children peanut as you introduced other foods? Tell me about this? [Practices]

© 2025 Samady W et al. *JAMA Network Open*.

6. At what age did you give peanut products?
7. How much did you give per feeding?
8. How long did you do this for?
9. Does your child eat peanut products now? [Practices]
10. Was this hard for you do to? What made it hard/easy?
11. Do you believe that there was an advantages to doing it? [Beliefs/Opinions]
12. Do you believe that there were any disadvantages? [Beliefs/Opinions]
13. Did you have any hesitations or concerns as you were going into it? [Beliefs/Opinions]

If participant answers NO (parent/caregiver has not heard of the guidelines):

1. If someone asked you to start feeding your child peanut products, how would you feel about this? [Beliefs/Opinions]
2. Would you want to do it? [Practices]
3. What would stop you from doing it, what would motivate you to do it?
4. Is this something you could easily get into your child's diet regularly? How would you go about it? [Practices]
5. Do you believe that there is an advantages to doing it? [Beliefs/Opinions]
6. Do you believe that there is any disadvantages? [Beliefs/Opinions]

Infant Atopy History and Family History Questions:

1. Are there members of your family who have food allergies? (if not already discussed)

   Probe: How did this influence how you fed your child foods? [Belief/Opinions and Practices]
2. Did your baby develop any food allergies? (if not already discussed)
3. Does your baby have eczema?

   Probe: did this affect how you fed your baby? [Knowledge]

© 2025 Samady W et al. *JAMA Network Open*.

eFigure. Recruitment Flow Diagram

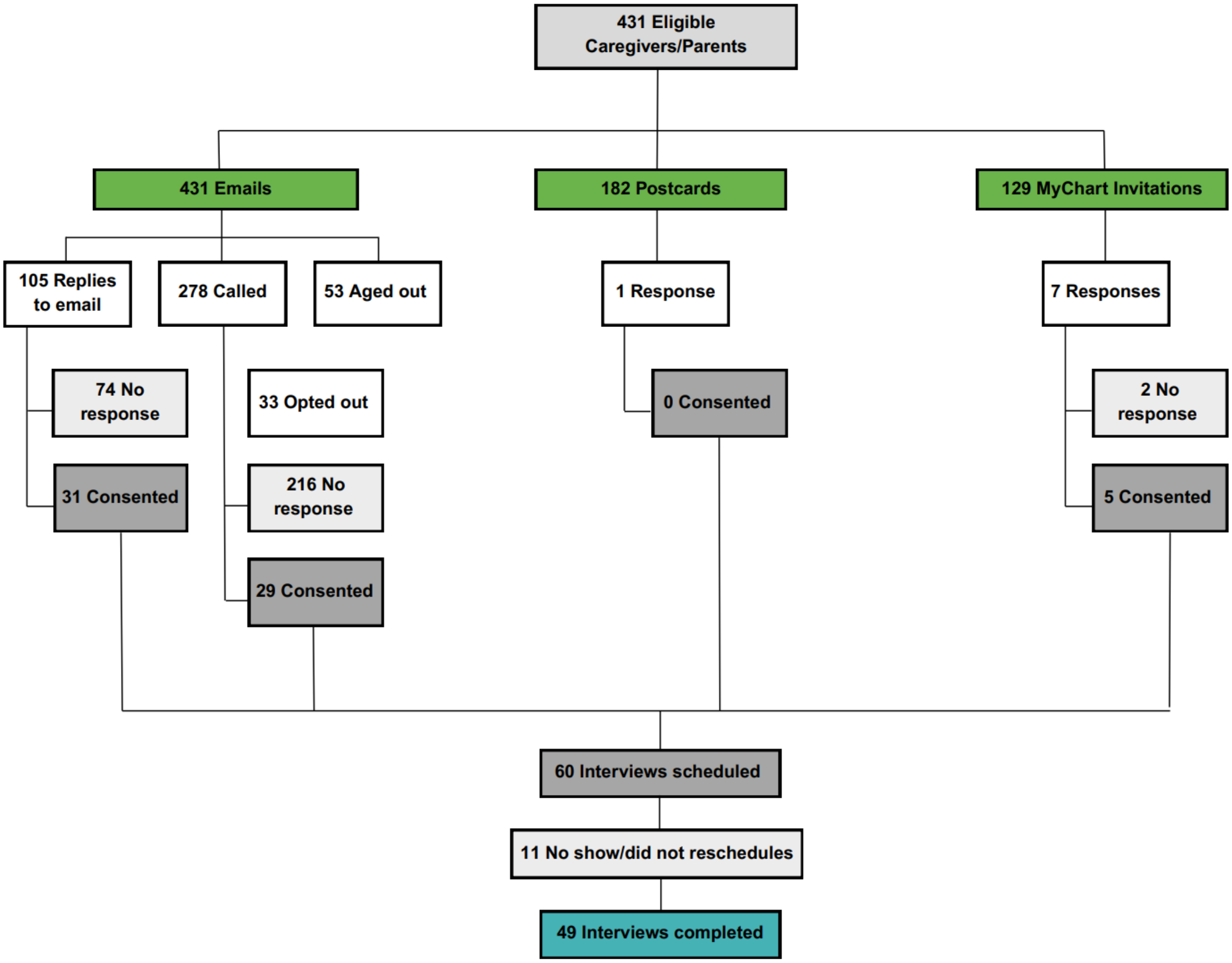

© 2025 Samady W et al. *JAMA Network Open*.

Focused Recruitment Procedure

The goal of this study was to obtain qualitative data from a diverse group of parents. Prior to scheduling interviews, participants completed a 20-question survey including sociodemographic data including race, ethnicity, insurance type, education level, family structure, child age, relationship to child, atopic disease in their family, and some diet related questions. Some information including age of their child, diagnoses, and research participation was validated using the electronic medical record.

During our initial recruitment, survey responses were gathered but all interested participants were invited to complete an interview regardless of their survey responses. As the major themes began to emerge and we felt that we were nearing saturation, the study team analyzed what types of participants were included in the 35 interviews we had completed at that time. We felt we had adequate representation from all race/ethnic groups, different education levels, and the presence and absence of atopic disease. However, we only have a few single parent families and almost two-thirds of the sample had private insurance.

Thus, we continued to recruit but this time we evaluated the 20-question survey and the EMR records initially and only extended interviews to parents who had a child with Medicaid insurance and/or parents who identified as single-parents. We continued to conduct interviews and thematic analysis until we felt we had more representation from these parents and felt we had achieved saturation within these groups as well (Table 1).

Sub-analysis of Major Themes

A sub-analysis evaluating several variables including private/public insurance, parental education, and family structure was conducted to identify if specific themes were more prominent within the subgroups. Using document memos in MaxQDA, each interview was summarized in general. After themes were

defined, each interview was evaluated in regards to major and minor themes and this was added to the document memos. Using the document variables feature in MaxQDA, interviews were selected based on the variable of interest. For example, all interviews parents who identified as single parent households were highlighted and reviewed. This review was done by evaluating the types of statements that were made as well as the frequency in which themes emerged in different groups.

eTable: Quantification of Major Themes

**Major Theme**	**Participants N = 49 (%)**
EP was to prevent peanut allergy	32 (65)
EP evaluates if infant is allergic to peanut (reaction)	23 (47)
Family history of food allergy was the main risk factor	31 (63)
EP was viewed positively	43 (88)
Fear of an allergic reaction served as a barrier to EP	10 (20)
Parents had mixed feelings about EP's overall benefit	17 (35)
The pediatrician was the primary source of EP knowledge	34 (69)

© 2025 Samady W et al. *JAMA Network Open*.

eAppendix 2. Supplemental Results

The subgroup analysis showed that the major and minor themes existed in all groups. While we had a high amount of awareness in our sample, there was a wide variation on how the EPPI guidelines were interpreted by the parents. This existed across subgroups. We did find, however, that parents who were unaware of the guidelines were more likely to have less than a bachelor's degree and/or have infants with public insurance. Fear of an allergic reaction existed in all education groups but was more common amongst parents who had less than a bachelor's degree. Additionally, family history as the main risk factor for food allergy was endorsed more frequently amongst parents with infants on public insurance. Requesting more information about the science behind EPPI was seen in both parents with public and private insurance for their infants but was more common amongst those parents who had a bachelor's degree or higher.

© 2025 Samady W et al. *JAMA Network Open*.

eREFERENCES

1. Ajzen I. The theory of planned behavior. *Organ Behav Hum Decis Process*. Dec 1991 1991;50(2):179-211. doi:10.1016/0749-5978(91)90020-T

2. Walsh A, Edwards H, Fraser J. Attitudes and subjective norms: determinants of parents' intentions to reduce childhood fever with medications. *Health Educ Res*. Jun 2009;24(3):531-45. doi:10.1093/her/cyn055

3. Ismail TA, Muda WM, Bakar MI. Intention of pregnant women to exclusively breastfeed their infants: The role of beliefs in the theory of planned behaviour. *J Child Health Care*. Jun 2014;18(2):123-32. doi:10.1177/1367493512473857

4. Walsh A, Kearney L, Dennis N. Factors influencing first-time mothers' introduction of complementary foods: a qualitative exploration. *BMC Public Health*. Sep 22 2015;15:939. doi:10.1186/s12889-015-2250-z

© 2025 Samady W et al. *JAMA Network Open*.
