# Supplementary material for: Parental Understanding and Implementation of Early Peanut Introduction
Source: JAMA Netw Open. 2025 Dec 18;8(12):e2550915. doi: 10.1001/jamanetworkopen.2025.50915 (PMC12715651; doi:10.1001/jamanetworkopen.2025.50915)
Supplement: Supplement 2. — Data Sharing Statement [file jamanetwopen-e2550915-s002.pdf]

Data Sharing Statement

Samady. Parental Understanding and Implementation of Early Peanut Introduction. *JAMA Netw Open*. Published December 18, 2025. doi:10.1001/jamanetworkopen.2025.50915

Data
Data available: Yes
Data types: Deidentified participant data
How to access data: waheeda.samady@northwestern.edu all interviews can be made avaialbe. other items are already available.
When available: beginning date: 07-01-2026

Supporting Documents
Document types: None

Additional Information
Who can access the data: Researchers whose proposed use of the data has been approved
Types of analyses: any purpose
Mechanisms of data availability: with a signed data access agreement
